# Factors Associated with Systemic Lupus Erythematosus-Associated Interstitial Lung Disease and Clinical Outcomes: A Systematic Review and Meta-Analysis

**DOI:** 10.3390/medicina62091673

**Published:** 2026-08-31

**Authors:** Mislav Radić, Petra Šimac Prižmić, Tina Bečić, Hana Đogaš, Josipa Radić, Damir Fabijanić, Andrea Gelemanović, Dijana Perković

**Affiliations:** 1 Division of Rheumatology, Allergology and Clinical Immunology, Department of Internal Medicine, Center of Excellence for Systemic Sclerosis in Croatia, University Hospital of Split, 21000 Split, Croatia; psimac@kbsplit.hr (P.Š.P.); dperkov@kbsplit.hr (D.P.); 2 School of Medicine, University of Split, 21000 Split, Croatia; dfabijan@mefst.hr; 3 Cardiovascular Disease Department, University Hospital of Split, 21000 Split, Croatia; tbecic@kbsplit.hr; 4 Department of Neurology, University Hospital of Split, 21000 Split, Croatia; hdogas@kbsplit.hr; 5 Division of Nephrology, Dialysis and Arterial Hypertension, Department of Internal Medicine, University Hospital of Split, 21000 Split, Croatia; 6 Mediterranean Institute for Life Sciences, University of Split, 21000 Split, Croatia; andrea.gelemanovic@gmail.com

**Keywords:** systemic lupus erythematosus, interstitial lung disease, associated factors, Raynaud phenomenon, prognosis, pulmonary fibrosis

## Abstract

*Background and Objectives*: Interstitial lung disease (ILD) is an uncommon but clinically important manifestation of systemic lupus erythematosus (SLE). We aimed to identify factors associated with SLE–ILD, evaluate factors associated with clinical outcomes in established disease, and quantitatively synthesize comparable data. *Materials and Methods*: PubMed, Web of Science, Scopus, and the Cochrane Central Register of Controlled Trials were searched from inception through July 2026. English-language observational studies of adults with SLE reporting factors associated with ILD occurrence or outcomes in established SLE–ILD were eligible. Random-effects meta-analyses were conducted when at least three studies reported comparable data. *Results*: Eight observational studies were included. Across five studies, patients with SLE–ILD were older than those without ILD (mean difference 8.49 years, 95% confidence interval [CI] 5.52–11.46; *p* < 0.0001; I^2^ = 66.9%). Across three studies, SLE–ILD was associated with higher odds of Raynaud phenomenon (odds ratio, 3.01; 95% CI, 1.49–6.09; *p* = 0.0022; I^2^ = 28.8%). Other study-level associations included smoking, serositis, features of overlap connective tissue diseases, selected autoantibodies, and Krebs von den Lungen-6 (KL-6). In established SLE–ILD, baseline forced vital capacity and cohort-specific clinical and imaging features were associated with outcomes, while population-based cohorts linked ILD with increased mortality. *Conclusions*: Older age and Raynaud phenomenon were the factors most consistently associated with SLE–ILD across the available comparative studies. Because most included studies were observational, these findings should be interpreted as associations rather than evidence of causality. Prognostic evidence remains limited and heterogeneous.

## 1. Introduction

Systemic lupus erythematosus (SLE) is a chronic autoimmune disease with heterogeneous, multiorgan involvement. Pleuropulmonary manifestations include pleuritis and pleural effusion, acute lupus pneumonitis, diffuse alveolar hemorrhage, pulmonary vascular disease, shrinking lung syndrome, and interstitial lung disease (ILD) [1,2,3]. Although ILD is less common in SLE than in systemic sclerosis or mixed connective tissue diseases (MCTDs), it is clinically significant because chronic parenchymal injury can lead to functional impairment and increased mortality [4,5,6]. Reported prevalence varies by population and method of ascertainment: administrative and registry studies generally report lower estimates than cohort studies that use systematic high-resolution computed tomography (HRCT) [3,4,5,6,7,8].

Pulmonary vascular involvement represents another clinically important pulmonary manifestation of SLE. Pulmonary hypertension (PH), including pulmonary arterial hypertension (PAH), may coexist with parenchymal lung disease and may adversely affect cardiopulmonary outcomes. Importantly, PAH and PH associated with ILD represent distinct pulmonary vascular phenotypes and should not be considered interchangeable [9,10].

The pathogenesis of SLE-associated ILD (SLE–ILD) remains incompletely understood. Proposed mechanisms include type I interferon–driven immune activation, immune complex deposition, neutrophil-mediated epithelial injury, and dysregulated repair, which together lead to fibrotic remodeling. These mechanisms are not specific to SLE and may be modified by features of overlap connective tissue disease, smoking, autoantibody profiles, and the radiologic pattern of lung injury [11,12].

Clinical studies have associated SLE–ILD with older age, Raynaud phenomenon (RP), serositis, myositis or overlap features, anti-U1 ribonucleoprotein and anti-Ro52 antibodies, and biomarkers of epithelial injury such as Krebs von den Lungen-6 (KL-6) [1,13,14]. However, reported associations are derived mainly from retrospective cohort studies and differ in population selection, ILD definitions, and statistical adjustment. Prediction models have recently been developed, but their variables and validation strategies are heterogeneous [15,16,17].

Prognostic evidence is even more limited. Multicenter SLE–ILD cohorts have evaluated pulmonary function, HRCT patterns, progression, transplantation-free survival, and mortality [18,19]. In contrast, nationwide administrative cohorts have examined the effect of ILD on survival in broader SLE populations [7,13]. Previous reviews have generally addressed pulmonary manifestations of SLE as a whole or compared early- and late-onset disease rather than distinguishing factors associated with ILD occurrence from determinants of outcome after ILD is established [20,21].

Distinguishing these two domains is clinically relevant because factors associated with prevalent SLE–ILD are not necessarily the same as those that determine disease progression or mortality once ILD is established. The present systematic review and meta-analysis therefore aimed to synthesize evidence on (1) factors associated with the presence of SLE–ILD and (2) factors associated with clinical outcomes in established SLE–ILD.

## 2. Materials and Methods

### 2.1. Study Design and Protocol Status

This systematic review and meta-analysis were conducted in accordance with the Preferred Reporting Items for Systematic Reviews and Meta-Analyses (PRISMA) 2020 statement [22]. The study protocol was prospectively registered in the International Prospective Register of Systematic Reviews (PROSPERO) (registration number CRD420261446107). The objectives were to identify and synthesize (1) factors associated with the presence of SLE–ILD and (2) factors associated with progression, transplantation-free survival, or mortality in established SLE–ILD. These domains were analyzed separately to avoid conceptual overlap.

### 2.2. Literature Search Strategy

A systematic literature search was conducted in PubMed, Web of Science Core Collection, Scopus, and the Cochrane Central Register of Controlled Trials (CENTRAL) via the Cochrane Library. The search covered all records from database inception through 1 July 2026. All strategies combined SLE and ILD concepts; association/prognostic terms were also included where documented in the database-specific strategy. Only English-language articles were eligible, and no date restriction was applied. Reference lists of eligible studies were screened manually. Full database-specific search strategies, search dates, and numbers of retrieved records are provided in Supplementary Material S1 (Table S1). The completed PRISMA 2020 Checklist is provided in Supplementary Material S2.

### 2.3. Eligibility Criteria

Studies were eligible if they included adults (≥18 years) with SLE, as diagnosed by the study authors using established classification criteria or validated administrative definitions, and evaluated ILD that was either prevalent or incident. Eligible observational designs included prospective or retrospective cohort studies, case–control studies, cross-sectional studies, and case–cohort studies. Studies had to report either (1) factors associated with the presence of SLE-associated ILD in comparative SLE populations or (2) factors associated with clinical outcomes in established SLE–ILD. ILD was accepted when defined by HRCT, multidisciplinary clinical diagnosis, or prespecified administrative codes for ILD or pulmonary fibrosis. Administrative definitions were accepted as reported by the original authors, with potential misclassification considered during risk-of-bias assessment.

Case reports, case series with fewer than 10 participants, reviews, editorials, conference abstracts, pediatric-only studies, non-SLE populations, overlapping cohorts without extractable SLE-specific data, and studies without relevant or interpretable data on ILD occurrence or prognosis were excluded. Studies of idiopathic pulmonary fibrosis or other ILD without an extractable SLE population were also excluded.

Outcomes were prespecified as (1) prevalent or incident ILD in patients with SLE and (2) ILD progression, pulmonary function decline, transplantation-free survival, all-cause or respiratory mortality, or other clearly defined outcomes in established SLE–ILD. Studies were grouped into these two domains.

### 2.4. Study Selection and Literature Processing

**Figure 1:**
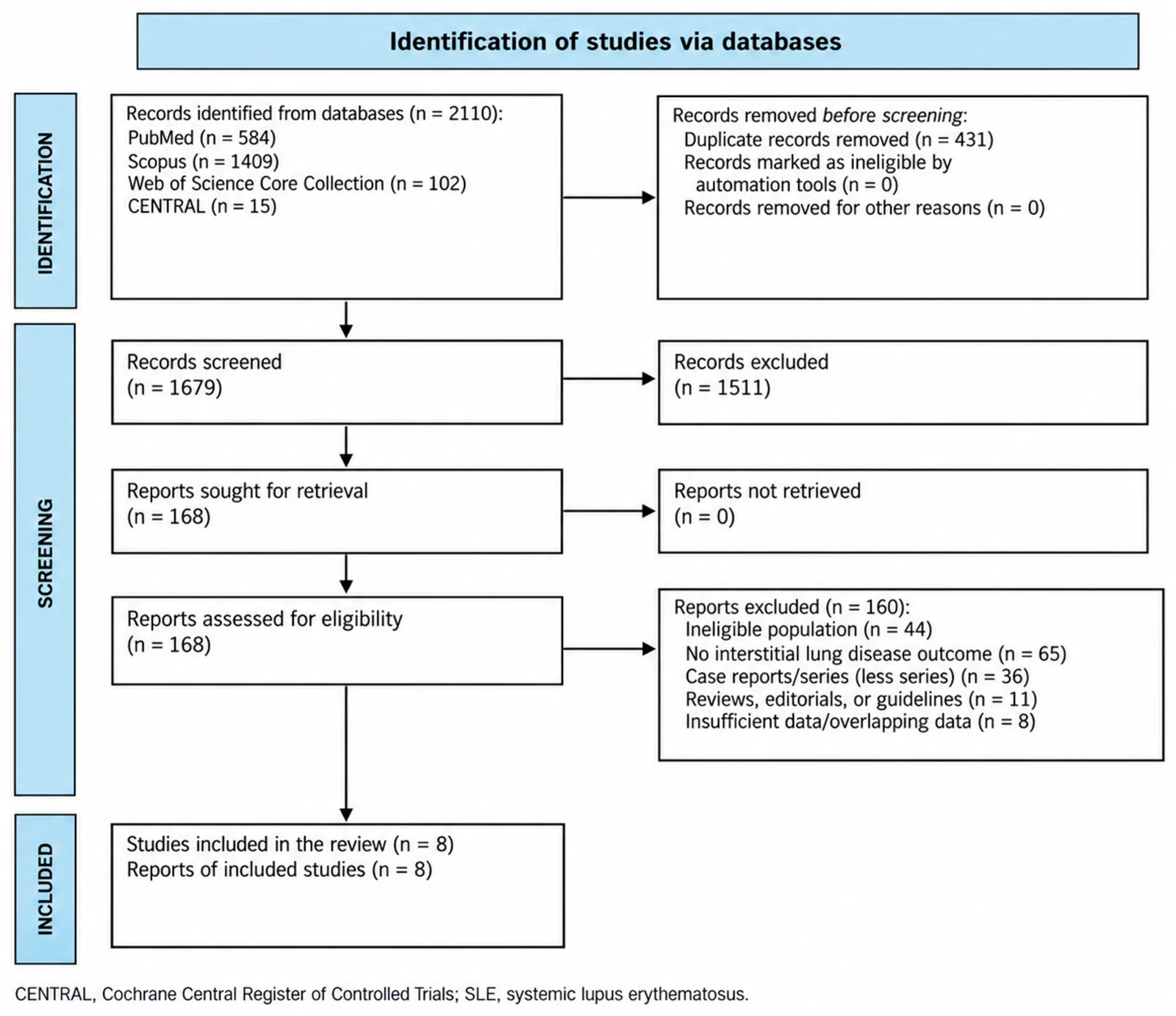
PRISMA 2020 flow diagram of study selection. Eight observational studies were included in the systematic review; five contributed to the age meta-analysis and three to the Raynaud phenomenon meta-analysis.

All retrieved records were compiled and deduplicated. Two reviewers (P.Š.P. and T.B.) independently screened titles and abstracts and subsequently assessed potentially eligible full texts. Disagreements were resolved through discussion and consensus, or through consultation with a third reviewer (M.R.). The study-selection process is shown in the PRISMA 2020 flow diagram (Figure 1).

### 2.5. Data Extraction

Two investigators (P.Š.P. and T.B.) independently extracted the first author, publication year, country, study design, population and sample size, ILD definition, assessed factors, effect estimates, and clinical outcomes. For quantitative synthesis, group sizes, means and standard deviations, medians and interquartile ranges, and 2 × 2 event counts were extracted when available. For studies reporting separate training and validation cohorts, only the training cohort was included in the quantitative synthesis to avoid participant duplication. Discrepancies were resolved by consensus.

### 2.6. Risk of Bias Assessment

Risk of bias was independently assessed by two reviewers using a domain-based approach informed by the Newcastle–Ottawa Scale (NOS). The domains were participant selection, exposure or outcome ascertainment, control of confounding, and follow-up or selective reporting. For prediction-model studies, the same domains were applied to participant selection, predictor and outcome ascertainment, modeling or confounding, and reporting. Overall risk was rated low when all domains were low, moderate when one or more domains raised some concern but none was high, and high when at least one domain indicated a serious risk of bias (low risk of bias: 7–9 stars; moderate risk of bias: 5–6 stars; high risk of bias: ≤4 stars). Disagreements were resolved by consensus. Results are reported in Section 3.3.

### 2.7. Data Synthesis and Statistical Analysis

We grouped factors associated with SLE–ILD and clinical outcomes into demographic, clinical, serological, biomarker, functional, and imaging categories for narrative synthesis. Random-effects meta-analyses were conducted when at least three independent studies reported comparable data on the same factor in patients with SLE–ILD and in those without ILD. For age, mean differences (MDs) and 95% confidence intervals (CIs) were pooled using the inverse-variance method; positive values indicated older age in the SLE–ILD group. When medians and interquartile ranges were reported, means and standard deviations were estimated using established methods. For RP, odds ratios (ORs) and 95% CIs were pooled using the Mantel–Haenszel method, with a continuity correction of 0.5 for zero-event cells. Between-study variance (τ^2^) was estimated using restricted maximum-likelihood estimation. Heterogeneity was evaluated using I^2^, τ^2^, and Cochran’s Q test. Two-sided *p* values < 0.05 were considered statistically significant. Analyses were performed in R version 4.5.1 (R Foundation for Statistical Computing, Vienna, Austria) [23], using the meta package version 8.2-1 [24]. Factors that did not meet pooling criteria were synthesized narratively and separated into factors associated with ILD occurrence and factors associated with clinical outcomes in established SLE–ILD. To evaluate how ILD ascertainment might affect the combined age estimate, we performed a sensitivity analysis excluding population-based studies that used administrative or hospital coding to identify ILD. This analysis therefore included only studies using HRCT-based clinical ascertainment of SLE–ILD. An analogous sensitivity analysis was not applicable to the RP meta-analysis because all studies contributing to that analysis used HRCT-based ILD ascertainment.

## 3. Results

### 3.1. Study Selection

The database searches identified 2110 records: 584 from PubMed, 102 from Web of Science, 1409 from Scopus, and 15 from CENTRAL. After removing 431 duplicate records, 1679 records underwent title and abstract screening, of which 1511 were excluded. Full texts were sought and retrieved for 168 reports. After full-text assessment, 160 reports were excluded: non-SLE population (*n* = 48), no ILD outcome (*n* = 45), case reports or small case series (*n* = 38), reviews, editorials, or guidelines (*n* = 21), and insufficient risk-factor or outcome data (*n* = 8). Eight observational studies were included in the qualitative synthesis. Five contributed to the age meta-analysis, and three to the RP meta-analysis (Figure 1).

### 3.2. Characteristics of Included Studies

The included studies were published from 2019 to 2026 and originated from Australia, Canada, China, France, Japan, and Turkey (Table 1). Designs included nationwide or population-level cohorts, hospital-based cohorts, multicenter SLE–ILD cohorts, a case–control study, a case–cohort analysis, and a model-development and validation study. ILD was identified using HRCT or a multidisciplinary evaluation in clinical cohorts, and via administrative coding in population-based studies. Sample sizes ranged from 55 patients with SLE-associated interstitial pneumonia to 10,460 patients in a baseline nationwide SLE cohort [7,8,13,15,18,19,25,26].

### 3.3. Risk of Bias

One study was judged to have an overall low risk of bias, and seven had a moderate risk; none was judged high risk (Table 2). The most frequent concerns were residual confounding, retrospective participant selection, and reliance on administrative coding or model-development procedures. Outcome ascertainment and follow-up or reporting were generally adequate.

### 3.4. Meta-Analysis

**Figure 2:**
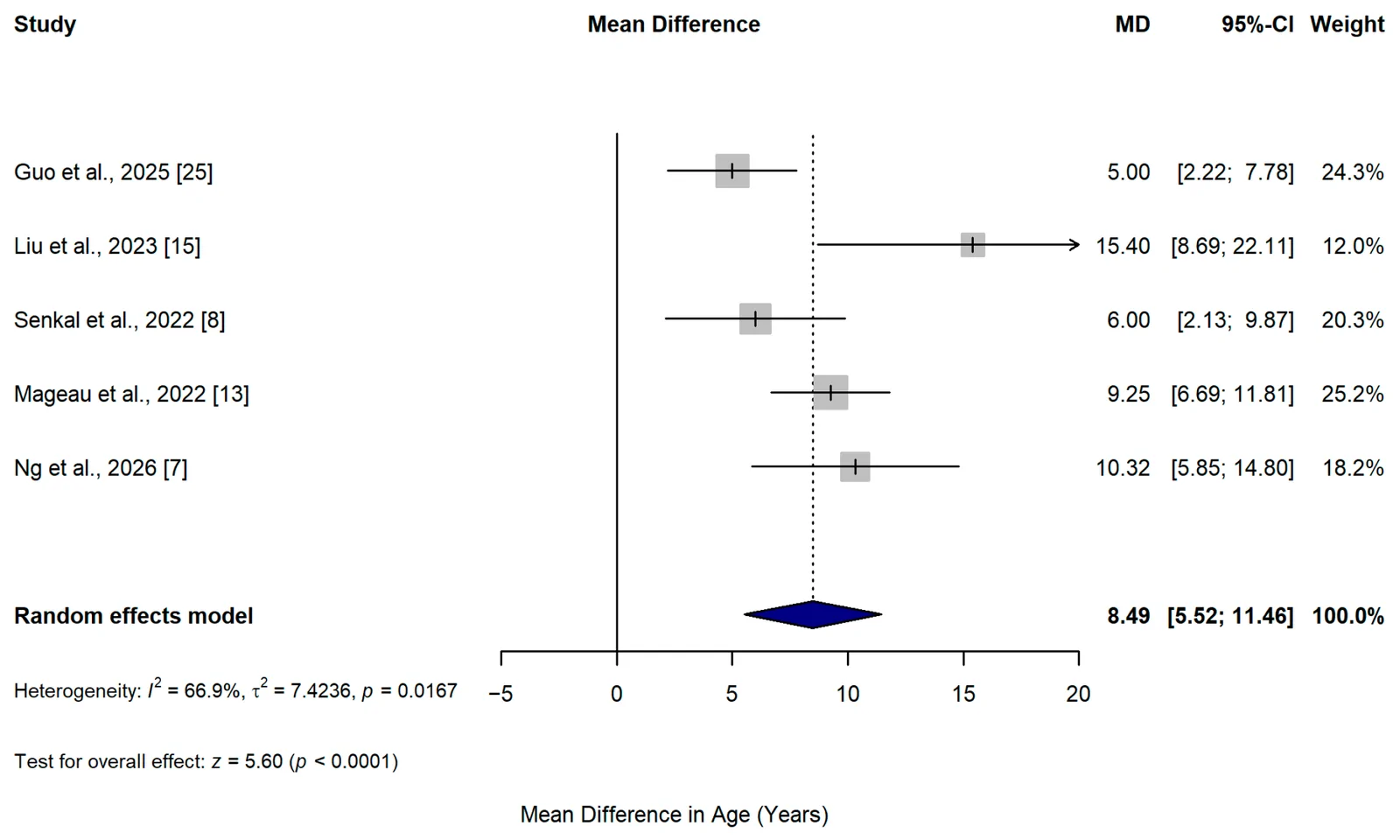
Forest plot of the mean difference in age between patients with SLE–ILD and SLE without ILD [7,8,13,15,25]. Positive values indicate older age in the SLE–ILD group. A random-effects inverse-variance model with restricted maximum-likelihood estimation was used.

**Figure 3:**
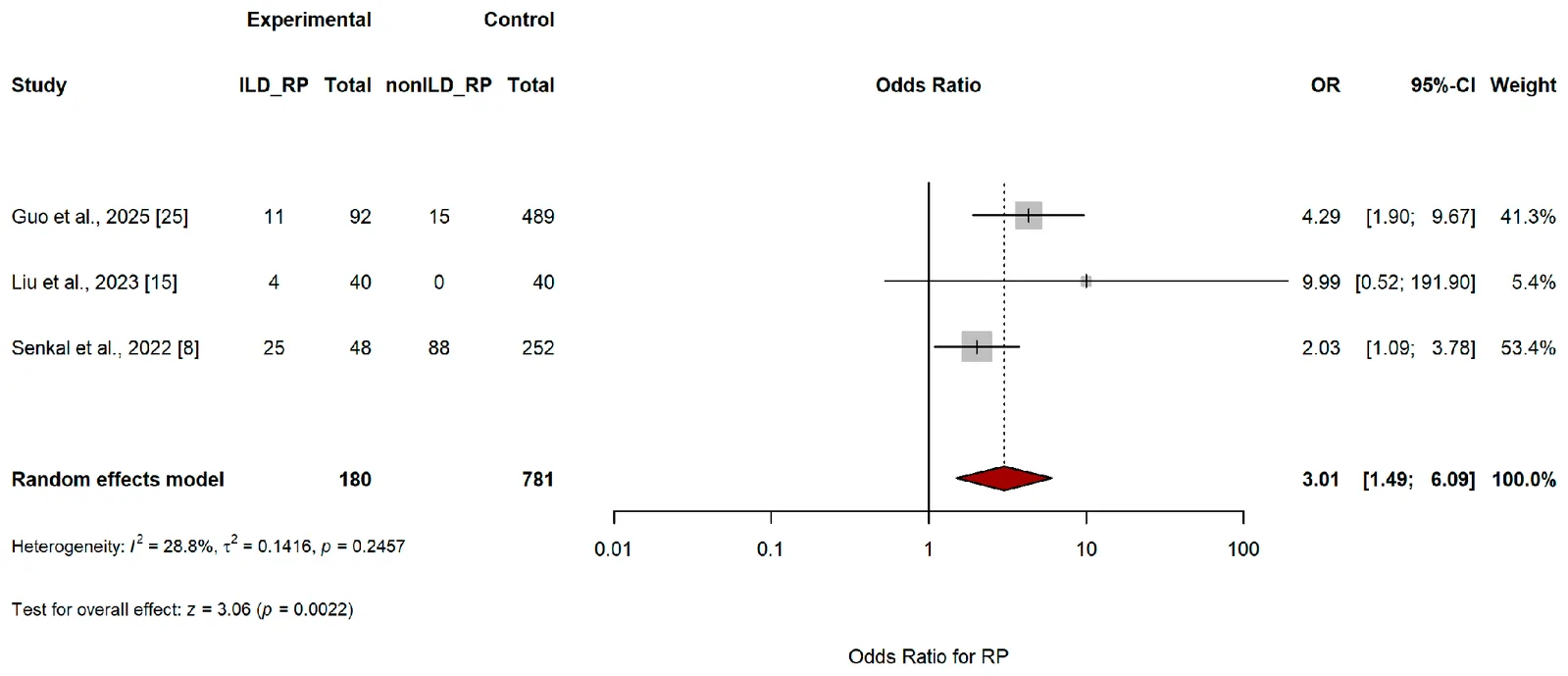
Forest plot of the association between Raynaud phenomenon and SLE–ILD [8,15,25]. Odds ratios greater than 1 indicate higher odds of Raynaud phenomenon in patients with SLE–ILD. A random-effects Mantel–Haenszel model with restricted maximum-likelihood estimation was used.

Five studies comprising 13,275 participants were included in the age analysis. Patients with SLE–ILD were, on average, 8.49 years older than those without ILD (MD 8.49 years, 95% CI 5.52–11.46; *p* < 0.0001), with substantial heterogeneity (I^2^ = 66.9%; τ^2^ = 7.4236; Cochran’s Q *p* = 0.0167) (Figure 2). Three studies comprising 961 participants were included in the RP analysis. SLE–ILD was associated with higher odds of RP (OR 3.01, 95% CI 1.49–6.09; *p* = 0.0022), with low heterogeneity (I^2^ = 28.8%; τ^2^ = 0.1416; Cochran’s Q *p* = 0.2457) (Figure 3). Because the available comparative evidence included predominantly retrospective cohort, case–control, and cross-sectional studies, these pooled estimates describe associations with prevalent SLE–ILD rather than causal risk factors for incident disease. Pooled results are summarized in Table 3.

**Figure 4:**
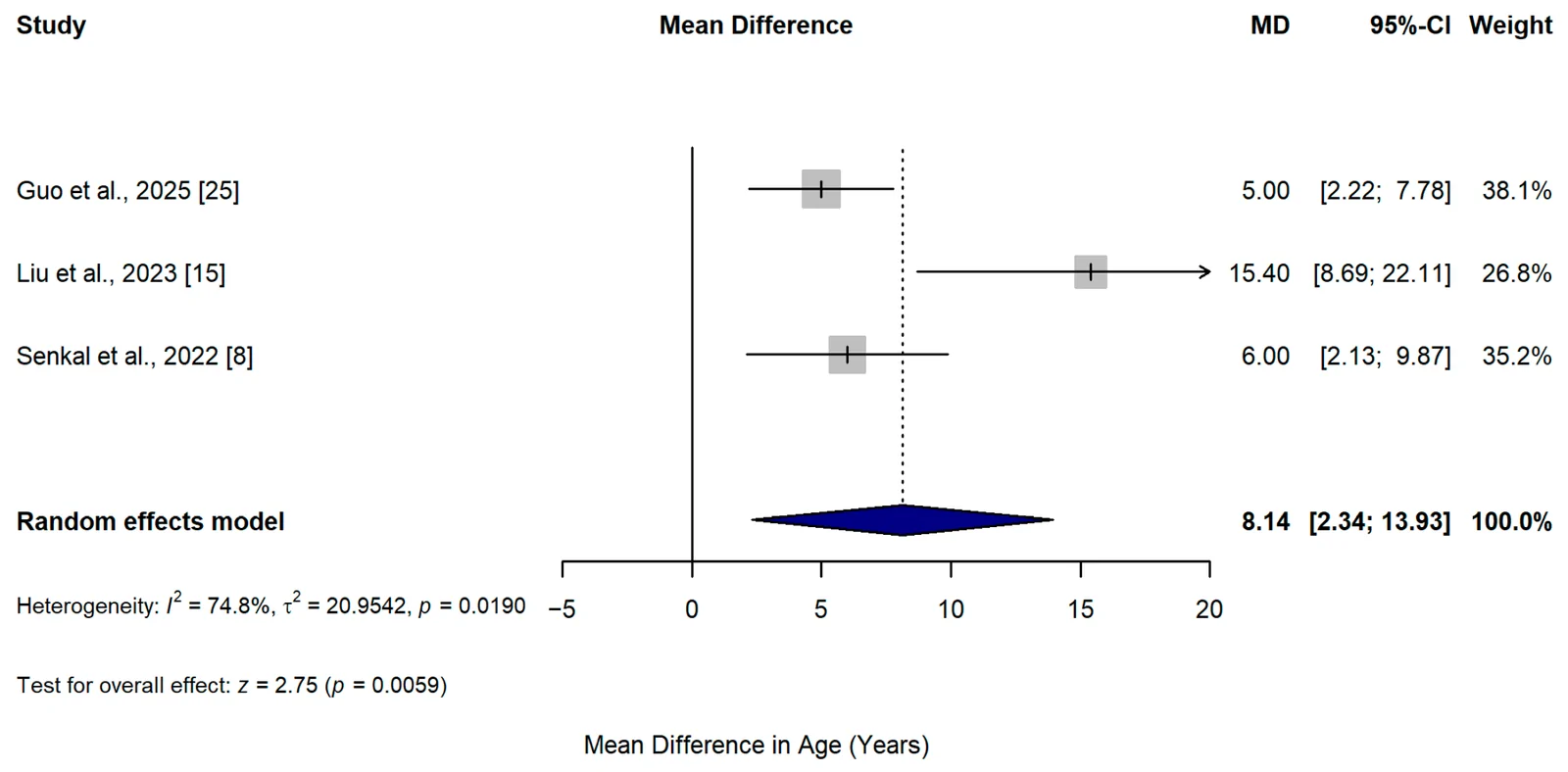
Sensitivity analysis of the mean age difference between patients with SLE–ILD and those with SLE without ILD, including only studies that used HRCT-based clinical ascertainment of ILD [8,15,25].

In a sensitivity analysis excluding population-based studies that identified ILD using administrative or hospital coding, three HRCT-based studies remained in the age meta-analysis. The pooled association persisted: patients with SLE–ILD were, on average, 8.14 years older than those with SLE without ILD (MD 8.14 years, 95% CI 2.34–13.93; *p* = 0.0059). Between-study heterogeneity remained substantial (I^2^ = 74.8%; τ^2^ = 20.9542; Cochran’s Q *p* = 0.0190) (Figure 4).

### 3.5. Factors Associated with the Presence of SLE–ILD

Beyond age and RP, individual studies reported additional associations. In the Australian population cohort, older age, smoking, and serositis were independently associated with ILD [7]. In the French nationwide cohort, ILD was more frequent in patients with another systemic autoimmune disease, particularly Sjögren syndrome or systemic sclerosis [13]. In the Turkish cohort, patients with SLE–ILD were more likely to have arthritis, serositis, RP, myositis, and anti-topoisomerase I antibodies [8]. Guo et al. identified RP, pulmonary artery systolic pressure, serositis, C-reactive protein, anti-U1 ribonucleoprotein (anti-U1RNP) and anti-Ro52 antibodies, age, and disease duration as model features associated with SLE–ILD [25]. Liu et al. reported independent associations with age, female sex, renal involvement, complement C3, immunoglobulin M, and anti-U1RNP antibodies [15]. In the Canadian case–cohort analysis, elevated baseline KL-6 was associated with subsequent ILD and/or myositis; this combined endpoint was not treated as an ILD-specific estimate [26]. Selected adjusted effect estimates are summarized in Table 4.

Disease activity was assessed in only a subset of the included studies and was not reported consistently. Enomoto et al. reported a median Systemic Lupus Erythematosus Disease Activity Index 2000 (SLEDAI-2K) score of 12 at the time of ILD diagnosis and found higher disease activity in acute/subacute interstitial pneumonia than in chronic interstitial pneumonia, although SLEDAI-2K was not associated with survival [18]. Liu et al., in contrast, observed lower SLEDAI scores in patients with SLE–ILD than in SLE controls without ILD [15]. Guo et al. included SLEDAI-2K in their predictive analyses, although it was not among the final overlapping critical features [25]. Overall, current evidence does not show a consistent relationship between global SLE activity and SLE–ILD.

### 3.6. Factors Associated with Clinical Outcomes

In the French multicenter cohort of 89 patients with established SLE-associated ILD, higher baseline forced vital capacity (FVC) was associated with improved transplantation-free survival and reduced risk of ILD progression. Cutaneous manifestations and RP were also associated with better transplantation-free survival, whereas no significant associations were observed for age, smoking status, diffusing capacity for carbon monoxide (DLCO), or HRCT pattern [19]. In the Japanese multicenter cohort, current smoking, thrombocytopenia, anti-double-stranded DNA titer, and the HRCT pattern were independently associated with prognosis [18]. Population-based studies addressed a different question: the effect of ILD on mortality in broader SLE populations. In France, ILD was independently associated with mortality after multivariable adjustment [13]. In Australia, the mortality rate among patients with SLE–ILD was higher than among patients with SLE without ILD [7]. These within-ILD prognostic findings and population-level mortality comparisons are distinguished in Table 5.

## 4. Discussion

This systematic review and meta-analysis found that patients with SLE–ILD were, on average, 8.49 years older than those with SLE without ILD and had approximately threefold higher odds of RP. Heterogeneity was substantial for age and low for RP. These pooled findings identify two readily recognizable markers of the SLE–ILD phenotype but do not establish causality or justify universal HRCT screening. Other associations—including smoking, serositis, overlap connective tissue diseases, anti-U1RNP and anti-Ro52 antibodies, and KL-6—were supported by individual studies with heterogeneous designs and could not be pooled [7,8,13,15,18,19,25,26].

Prognostic evidence should be interpreted separately from risk-factor evidence. In established SLE–ILD cohorts, baseline FVC and selected clinical, laboratory, and HRCT features were associated with survival or progression [18,19]. In contrast, population studies by Mageau et al. and Ng et al. assessed mortality associated with ILD in broader SLE populations [7,13]. Thus, ILD itself is associated with increased mortality in SLE, but evidence on determinants of outcome among patients who already have SLE–ILD remains limited.

Pulmonary vascular involvement may provide additional context for the clinical heterogeneity of SLE–ILD. Guo et al. identified elevated pulmonary artery systolic pressure (PASP) as a critical feature associated with SLE–ILD [25]. Deneuville et al. reported suspected PH on echocardiography in 16 of 89 patients (18%) with SLE–ILD at the time of ILD diagnosis; right-heart catheterization data were not available. PH was not associated with transplantation-free survival in their univariable analysis, although pulmonary hypertension was reported as a cause of respiratory failure leading to death in three patients [19].

Recent studies also indicate heterogeneity within SLE-PAH and connective tissue disease-associated pulmonary arterial hypertension (CTD-PAH) by autoantibody and inflammatory phenotypes. Baisya et al. identified distinct autoantibody-based clusters among patients with SLE-PAH, with differences in PAH severity and clinical outcomes [9]. Wu et al. identified vasculopathic, vasculitic, and mixed phenotypes among patients with CTD-PAH, which differed in inflammatory profiles, hemodynamic characteristics, treatment response, and survival [10]. Importantly, these findings provide complementary context regarding pulmonary vascular involvement but should not be interpreted as direct evidence for ILD-associated PH; notably, Wu et al. excluded patients with moderate or severe ILD [9,10].

The association with RP is consistent with the clustering of SLE–ILD with overlap connective tissue diseases and selected autoantibody profiles. A recently published meta-analysis of pulmonary complications in SLE likewise identified RP as one of the more consistent ILD-associated factors. However, the included populations and analytic framework differed from those of the present review [26]. Collectively, the evidence supports clinical vigilance in older patients and in those with RP, respiratory symptoms, overlap features, or suggestive serology. Pulmonary function testing and targeted HRCT may be appropriate when clinical suspicion is present, but evidence is insufficient to recommend universal HRCT screening.

Although HRCT remains the diagnostic gold standard, its use as a screening tool has not yet been standardized. Expert consensus statements increasingly emphasize a structured approach to assessing ILD associated with systemic autoimmune diseases, including baseline pulmonary function testing (PFT) and imaging triggered by respiratory symptoms or other SLE-related features suggestive of lung involvement [27,28]. After diagnosing SLE–ILD, assessing disease severity and the extent of pulmonary involvement is essential to guide therapeutic decisions. To improve comparability across studies and facilitate clinical decision-making, standardized terminology and imaging classification systems are needed [29]. Finally, the mortality signal observed in population-based studies underscores that SLE–ILD is not merely a radiographic finding but a determinant of long-term outcome. Early identification and multidisciplinary management, including close collaboration among rheumatologists, pulmonologists, and radiologists, are therefore crucial [30,31]. Based on current evidence, a risk-stratified screening algorithm for SLE patients—prioritizing initial HRCT and comprehensive PFT, including diffusing capacity for carbon monoxide—may be considered; however, prospective validation is required. SLE–ILD encompasses different radiologic patterns, most commonly non-specific interstitial pneumonia (NSIP), and often begins insidiously [32]. HRCT findings further illustrate the heterogeneity of SLE–ILD. Enomoto et al. reported an unclassifiable HRCT pattern in 54% of patients, with NSIP + organizing pneumonia (OP) accounting for 25% of the cohort, whereas Deneuville et al. reported NSIP with or without superimposed OP in 46.1% of patients [18,19]. Liu et al. observed a predominantly NSIP phenotype (90%), and Şenkal et al. similarly reported NSIP as the predominant pattern [8,15]. Collectively, these findings suggest that NSIP-spectrum abnormalities are common in SLE-ILD, although unclassifiable, indeterminate, OP, usual interstitial pneumonia (UIP), diffuse alveolar damage (DAD), and cystic/lymphocytic interstitial pneumonia (LIP) patterns may also occur. Thus, SLE–ILD should be considered radiologically heterogeneous rather than characterized by a single disease-specific HRCT pattern. International classification systems for ILD emphasize integrating radiologic and histopathologic patterns to improve diagnostic accuracy [31,32,33]. Many patients may initially be asymptomatic or report only mild dyspnea, which supports the possibility of a subclinical phase and reinforces the need for risk-based evaluation [34]. Pathophysiologically, SLE–ILD reflects persistent immune-mediated epithelial injury followed by dysregulated repair and fibrotic remodeling [35,36]. Chronic inflammation promotes extracellular matrix deposition, and histological findings most frequently demonstrate an inflammatory NSIP pattern, which is in concordance with radiologic findings [32]. Diagnosis requires careful exclusion of alternative causes, including infection and overlap connective tissue diseases.

Available evidence indicates substantial overlap but also differences in the relative distribution of HRCT patterns: NSIP predominates in systemic sclerosis and idiopathic inflammatory myopathies, whereas UIP is more frequent in rheumatoid arthritis; Sjögren disease-associated ILD is heterogeneous, with NSIP and UIP as major patterns and LIP occurring relatively more frequently than in other CTDs [37]. In inflammatory myopathies, the clinical course is further influenced by autoantibody phenotype, with anti-aminoacyl-tRNA synthetase (anti-ARS) antibodies more commonly associated with chronic ILD and anti-melanoma differentiation-associated gene 5 (anti-MDA5) antibodies with rapidly progressive ILD [38]. In contrast, the SLE studies included in our review show a heterogeneous radiological phenotype, often within the NSIP spectrum, with a notable occurrence of overlap CTD features. No HRCT pattern is specific to a single systemic autoimmune rheumatic disease, and radiological findings should be interpreted within the broader clinical and serological phenotype.

At a mechanistic level, SLE–ILD falls within the broader spectrum of connective tissue disease-associated ILD, in which immune dysregulation, epithelial injury, aberrant repair, and progressive extracellular matrix deposition interact [15,16,26,39]. Neutrophil activation and extracellular traps, type I interferon signaling, and shared profibrotic pathways provide biologically plausible links between systemic autoimmunity and pulmonary fibrosis. However, direct mechanistic evidence for SLE remains limited [18,40,41]. The association of anti-U1RNP, anti-Ro52, and anti-Ku antibodies with ILD risk aligns with data indicating that interferon-driven immune activation plays a central role in connective tissue disease-associated lung injury [1,42]. Sustained type I interferon signaling promotes autoreactive B-cell activation, immune complex formation, and persistent epithelial injury, potentially initiating the transition from inflammation to fibrosis [43]. Elevated KL-6 levels further support the concept of ongoing alveolar epithelial damage preceding overt structural remodeling [39,44]. In this context, epithelial injury may trigger fibroblast activation and extracellular matrix deposition, establishing a self-perpetuating profibrotic feedback loop, as described in other fibrosing autoimmune lung diseases [45,46]. In contrast, once ILD is established, prognosis appears to be driven less by baseline immunologic phenotype and more by the extent of irreversible structural damage. Reduced FVC and greater fibrotic burden on HRCT likely reflect advanced matrix accumulation and architectural distortion, markers of a fibrosis-dominated phase that may progress independently of systemic inflammatory activity. This pattern resembles the progressive fibrosing phenotype observed in other systemic autoimmune diseases [47]. Growing recognition of progressive fibrosing ILD has led to the identification of common clinical pathways among fibrosing pulmonary disorders [48]. Taken together, these findings support a conceptual model suggesting that SLE–ILD evolves from an early, immune-mediated phase to a later stage characterized by structural lung remodeling and functional decline. This transition may explain why predictors of ILD development differ from determinants of outcome once fibrotic disease is established.

Biomarker and diagnostic studies further support a multidimensional assessment rather than reliance on a single clinical feature. Cytokine profiles and epithelial injury biomarkers such as KL-6 may assist with phenotyping. At the same time, experience with interstitial pneumonia with autoimmune features and other systemic autoimmune diseases emphasizes the need for standardized terminology, multidisciplinary review, and integration of clinical, serological, functional, and radiological findings [21,27,29,35,39]. These sources provide contextual support but were not treated as additional SLE–ILD risk-factor cohorts in the present meta-analysis.

Treatment reporting was heterogeneous across the included studies. Corticosteroids were commonly used for established SLE–ILD, often in combination with immunosuppressive therapy. Enomoto et al. reported corticosteroid use in 51/55 patients, with additional immunosuppression in 22 [18]. Deneuville et al. reported use of corticosteroids and several immunosuppressive agents, including mycophenolate mofetil, cyclophosphamide, rituximab, and azathioprine, while Şenkal et al. reported more frequent historical mycophenolate mofetil (MMF) exposure in the ILD group than in the non-ILD group [8,19]. Because treatment allocation was non-randomized and treatment reporting was not standardized, the included studies do not permit conclusions regarding comparative therapeutic efficacy. Current management guidance for autoimmune rheumatic disease-associated ILD remains largely based on extrapolation across diseases and on characterization of the progressive-fibrosing phenotype [42,43]. Glucocorticoids and immunosuppressive agents are commonly used, while biologic or antifibrotic therapies may be considered in selected progressive cases, guided by the broader ILD phenotype and multidisciplinary assessment [1,2,42,49]. Antifibrotic efficacy has been demonstrated in progressive fibrosing ILDs as a group, but this does not establish the effectiveness of SLE-specific treatment [50]. Additional real-world evidence regarding nintedanib in CTD-ILD has recently been reported by Gkouvi et al. In a retrospective cohort of 60 patients with CTD-ILD, most of whom had systemic sclerosis, rheumatoid arthritis, or idiopathic inflammatory myopathy, mean FVC% did not change significantly during nintedanib treatment (66.5 ± 16.4 at baseline vs. 64.1 ± 19.0 during treatment; *p* = 0.090), while 96.6% of patients received concomitant immunosuppressive therapy. Dose reduction was required in 26.7% of patients and permanent discontinuation in 6.7%, with diarrhea being the most frequently reported adverse event (21.6%). These real-world findings provide additional information regarding the use and tolerability of nintedanib in CTD-ILD; however, because the cohort was heterogeneous and did not provide SLE-specific efficacy data, they should not be extrapolated as evidence of efficacy specifically in SLE–ILD [46].

Standardized radiologic classification and consistent outcome definitions are therefore essential for future SLE–ILD trials [50]. Broader SLE and connective tissue disease cohorts also illustrate the mortality burden associated with pulmonary involvement and underscore that prognostic estimates from other diseases should not be transferred uncritically to SLE–ILD [33]. Prospective SLE-specific studies are needed to determine whether early recognition of high-risk phenotypes improves long-term pulmonary outcomes.

Several limitations should be acknowledged. Clinical and methodological heterogeneity limited quantitative synthesis to age and RP. A key limitation of this review is not only the heterogeneity between studies but also the small and methodologically limited evidence base. Only eight observational studies met the eligibility criteria: five for the meta-analysis of age and three for Raynaud phenomenon. Seven of these studies were assessed as having a moderate overall risk of bias, with only one rated as low. Significant methodological concerns included residual confounding, retrospective participant selection in several studies, variations in ILD diagnosis, and heterogeneity in outcome assessment. Therefore, the pooled estimates should be interpreted with caution, as the small number of studies and their methodological limitations reduce precision and applicability and preclude causal interpretation.

ILD definitions ranged from HRCT-confirmed disease to administrative coding, and study designs included case–control studies, single- or multicenter cohorts, and nationwide registries. The age analysis showed substantial heterogeneity, and converting medians and interquartile ranges introduced uncertainty. The RP analysis involved only three studies; one used counts reconstructed from percentages, and a continuity correction was applied to address a zero-event cell. Other effect estimates could not be pooled because of differences in outcomes, effect measures, and covariate adjustment. Most studies were retrospective, so residual confounding and misclassification cannot be excluded. The small number of studies precluded formal assessment of publication bias and extensive subgroup analyses. A sensitivity analysis excluding studies that used administrative or hospital coding for age yielded a pooled estimate similar to the primary analysis; however, only three HRCT-based studies remained, the confidence interval widened, and substantial heterogeneity persisted. Therefore, this analysis supports the consistency of the direction and approximate magnitude of the age association but does not eliminate concerns about clinical and methodological heterogeneity or the limited evidence base. Finally, some included studies assessed combined or population-level outcomes rather than ILD-specific progression, limiting direct clinical inference for established SLE–ILD. Larger prospective studies using standardized definitions of SLE-ILD, consistent radiologic and clinical ascertainment, and harmonized outcome measures are needed to confirm these associations and clarify their clinical relevance.

## 5. Conclusions

Patients with SLE–ILD were, on average, 8.49 years older than patients with SLE without ILD and had approximately threefold higher odds of Raynaud phenomenon. These are the factors most consistently associated with SLE-associated interstitial lung disease across the available comparative evidence. Other clinical, serological, and biomarker associations are derived mainly from individual heterogeneous studies. Prognostic evidence in established SLE–ILD is limited, while population-based cohorts consistently indicate increased mortality when ILD is present. A risk-stratified approach to pulmonary evaluation and follow-up is reasonable, but screening and prognostic strategies require prospective validation using standardized definitions and outcomes.

## Figures and Tables

**Table 1:** Characteristics of the included studies.

Author (Year)	Country	Study Design	Population/Sample Size	ILD Definition	Key Variables	Main Outcomes
Ng et al. (2026) [7]	Australia	Population-level cohort	SLE *n* = 1854; matched controls *n* = 12,107	Hospital coding	Age, smoking, serositis	ILD incidence and mortality
Şenkal et al. (2022) [8]	Turkey	Hospital-based cohort	SLE *n* = 300; ILD *n* = 48	HRCT	Clinical features, PFT	ILD prevalence and lung function
Mageau et al. (2022) [13]	France	Nationwide cohort	Baseline SLE cohort *n* = 10,460; incidence cohort *n* = 31,029	ICD codes	Age and overlap autoimmune disease	ILD prevalence, incidence, and mortality
Enomoto et al. (2019) [18]	Japan	Multicenter retrospective cohort	SLE–ILD *n* = 55	HRCT and multidisciplinary review	Smoking, laboratory and HRCT features	Prognostic factors and survival
Guo et al. (2025) [25]	China	Retrospective model-development and validation study	Training *n* = 581 (included in the meta-analysis); test *n* = 86	HRCT-based diagnosis	Demographic, clinical, serologic, and laboratory features	Risk-prediction model
Deneuville et al. (2024) [19]	France	Multicenter retrospective cohort	SLE–ILD *n* = 89	HRCT	FVC, clinical and serologic features	Progression and transplantation-free survival
Liu et al. (2023) [15]	China	Case–control study	SLE–ILD *n* = 40; SLE without ILD *n* = 40	HRCT	Clinical, laboratory, and serologic factors	Associated factors and risk model
Cotton et al. (2022) [26]	Canada	Prospective case–cohort analysis	Parent cohort *n* = 551; analytic subcohort *n* = 72; 14 incident ILD/myositis cases	Chart-confirmed SDI definition	KL-6, anti-Ro52, anti-Ku, and other biomarkers	Combined incident ILD and/or myositis endpoint

Abbreviations: FVC, forced vital capacity; HRCT, high-resolution computed tomography; ILD, interstitial lung disease; PFT, pulmonary function testing; SDI, Systemic Lupus International Collaborating Clinics/American College of Rheumatology Damage Index; SLE, systemic lupus erythematosus.

**Table 2:** Risk-of-bias assessment of the included studies.

Study	Selection Bias	Exposure/Outcome Assessment	Confounding/Modeling	Follow-Up/Reporting	Overall Risk
Ng et al. (2026) [7]	Low	Low	Low	Low	Low
Şenkal et al. (2022) [8]	Moderate	Low	Moderate	Low	Moderate
Mageau et al. (2022) [13]	Low	Moderate	Moderate	Low	Moderate
Enomoto et al. (2019) [18]	Moderate	Low	Moderate	Low	Moderate
Guo et al. (2025) [25]	Moderate	Low	Moderate	Low	Moderate
Deneuville et al. (2024) [19]	4 Low	Low	Moderate	Low	Moderate
Liu et al. (2023) [15]	Moderate	Low	Low	Low	Moderate
Cotton et al. (2022) [26]	Moderate	Moderate	Moderate	Low	Moderate

**Table 3:** Summary of pooled meta-analysis results.

Outcome	Studies (*n*)	Effect	95% CI	*p* Value	Heterogeneity
Age	5 (13,275)	MD 8.49 years	5.52–11.46	<0.0001	I^2^ = 66.9%; τ^2^ = 7.4236; Q *p* = 0.0167
Raynaud phenomenon	3 (961)	OR 3.01	1.49–6.09	0.0022	I^2^ = 28.8%; τ^2^ = 0.1416; Q *p* = 0.2457

Abbreviations: CI, confidence interval; MD, mean difference; OR, odds ratio; Q, Cochran’s Q statistic; τ^2^, between-study variance; I^2^, inconsistency statistic.

**Table 4:** Selected adjusted associations with SLE–ILD occurrence or the combined ILD/myositis endpoint.

Study	Factor	Effect	95% CI	Outcome/Model
Ng 2026 [7]	Older age	OR 1.04	1.02–1.05	Incident ILD; multivariable logistic regression
Ng 2026 [7]	Smoking	OR 1.85	1.13–3.02	Incident ILD; multivariable logistic regression
Ng 2026 [7]	Serositis	OR 2.10	1.14–3.90	Incident ILD; multivariable logistic regression
Liu 2023 [15]	Anti-U1RNP positivity	OR 19.886	Not reported	SLE–ILD; multivariable logistic regression
Cotton 2022 [26]	Elevated KL-6	aHR 3.66	1.01–13.3	Combined incident ILD and/or myositis endpoint

Effect measures are presented as reported in the original studies and were not pooled because outcome definitions and adjustment strategies differed across studies. Abbreviations: aHR, adjusted hazard ratio; CI, confidence interval; ILD, interstitial lung disease; KL-6, Krebs von den Lungen-6; OR, odds ratio; SLE, systemic lupus erythematosus; U1RNP, U1 ribonucleoprotein.

**Table 5:** Study-specific prognostic associations in established SLE–ILD and mortality associated with ILD in population cohorts.

Study	Factor	Outcome	Effect	95% CI/*p*	Population
Deneuville 2024 [19]	Higher baseline FVC	Transplant-free survival and ILD progression	Significant association	HR not reported	Established SLE–ILD cohort
Deneuville 2024 [19]	Cutaneous manifestations	Transplantation-free survival	HR = 0.25	95% CI 0.09–0.70; *p* = 0.009	Established SLE–ILD cohort
Deneuville 2024 [19]	Raynaud phenomenon	Transplantation-free survival	HR = 0.33	95% CI 0.11–0.99; *p* = 0.049	Established SLE–ILD cohort
Enomoto 2019 [18]	Current smoking	Mortality	HR = 6.105	*p* = 0.027	Established SLE–ILD cohort
Enomoto 2019 [18]	Thrombocytopenia	Mortality	HR = 7.676	*p* = 0.010	Established SLE–ILD cohort
Enomoto 2019 [18]	Anti-dsDNA titer	Mortality	HR = 0.956	*p* = 0.027	Established SLE–ILD cohort
Enomoto 2019 [18]	NSIP + OP vs. NSIP	Mortality	HR = 0.089	*p* = 0.023	Established SLE–ILD cohort
Mageau 2022 [13]	Presence of ILD	All-cause mortality	aHR = 1.992	95% CI 1.420–2.794	Nationwide SLE cohort
Ng 2026 [7]	Presence of ILD	Mortality rate	MRR = 2.94	NR	SLE–ILD vs. SLE without ILD

Abbreviations: aHR, adjusted hazard ratio; anti-dsDNA, anti-double-stranded DNA; CI, confidence interval; FVC, forced vital capacity; HR, hazard ratio; ILD, interstitial lung disease; MRR, mortality rate ratio; NR, not reported; NSIP, nonspecific interstitial pneumonia; OP, organizing pneumonia; SLE, systemic lupus erythematosus.

## Data Availability

The study-level data supporting the pooled analyses are contained within the article and are available from the corresponding author upon reasonable request.

## Supplementary Materials

The following supporting information can be downloaded at https://www.mdpi.com/article/10.3390/medicina62091673/s1. Supplementary Material S1: Database-specific search strategies and search yields (Table S1). Supplementary Material S2: PRISMA 2020 Checklist.

## Abbreviations

The following abbreviations are used in this manuscript:

aHR	adjusted hazard ratio
CI	confidence interval
DLCO	diffusing capacity for carbon monoxide
FVC	forced vital capacity
HR	hazard ratio
HRCT	high-resolution computed tomography
ICD	International Classification of Diseases
ILD	interstitial lung disease
IQR	interquartile range
KL-6	Krebs von den Lungen-6
MD	mean difference
NOS	Newcastle–Ottawa Scale
OR	odds ratio
PAH	pulmonary arterial hypertension
PFT	pulmonary function testing
PH	pulmonary hypertension
PRISMA	Preferred Reporting Items for Systematic Reviews and Meta-Analyses
PROSPERO	International Prospective Register of Systematic Reviews
RP	Raynaud phenomenon
SLE	systemic lupus erythematosus
SLE–ILD	systemic lupus erythematosus-associated interstitial lung disease
