# Supplementary material for: Factors Associated with Systemic Lupus Erythematosus-Associated Interstitial Lung Disease and Clinical Outcomes: A Systematic Review and Meta-Analysis
Source: Medicina (Kaunas). 2026 Aug 31;62(9):1673. doi: 10.3390/medicina62091673 (PMC13609442; doi:10.3390/medicina62091673)
Supplement: Supplementary file 1 [file medicina-62-01673-s001.zip › medicina-4478671-supplementary.pdf]

Supplementary Table S1. Database-specific search strategies

Systematic review and meta-analysis of factors associated with and prognostic determinants of SLE-associated interstitial lung disease

Searches were conducted from database inception without date restrictions. English-language eligibility was applied during study selection. Counts shown are the records exported before cross-database deduplication. The PubMed search strategy was reconstructed from the documented search concepts and exported result set because the original search-history string was no longer available; the other strategies reproduce the recorded searches.

PubMed — last searched 1 July 2026; records retrieved: 584

```
("systemic lupus erythematosus"[Title/Abstract] OR "systemic lupus"[Title/Abstract] OR SLE[Title/Abstract]) AND ("interstitial lung disease"[Title/Abstract] OR ILD[Title/Abstract] OR "pulmonary fibrosis"[Title/Abstract] OR "interstitial pneumonia"[Title/Abstract]) AND (risk[Title/Abstract] OR risks[Title/Abstract] OR "risk factor"[Title/Abstract] OR "risk factors"[Title/Abstract] OR predictor*[Title/Abstract] OR determinant*[Title/Abstract] OR association*[Title/Abstract] OR prognos*[Title/Abstract] OR progression[Title/Abstract] OR survival[Title/Abstract] OR mortality[Title/Abstract])
```

Scopus — last searched 1 July 2026; records retrieved: 1,409

```
TITLE-ABS-KEY(("systemic lupus erythematosus" OR SLE) AND ("interstitial lung disease" OR ILD OR "pulmonary fibrosis" OR "interstitial pneumonia") AND (risk OR risks OR "risk factor" OR "risk factors" OR predictor OR predictors OR determinant OR determinants OR association OR associations OR prognosis OR prognostic OR progression OR survival OR mortality))
```

Web of Science Core Collection (Topic) — last searched 1 July 2026; records retrieved: 102

```
TS=(("systemic lupus erythematosus" OR "systemic lupus" OR SLE) AND ("interstitial lung disease" OR "interstitial lung diseases" OR ILD OR "pulmonary fibrosis" OR "interstitial pneumonia" OR "interstitial pneumonias" OR "interstitial pneumonitis" OR "diffuse parenchymal lung disease"))
```

CENTRAL via Cochrane Library — last searched 1 July 2026; records retrieved: 15

```
("systemic lupus erythematosus" OR SLE):ti,ab,kw AND ("interstitial lung disease" OR ILD OR "pulmonary fibrosis" OR "interstitial pneumonia"):ti,ab,kw AND (risk OR risks OR "risk factor" OR "risk factors" OR predictor OR predictors OR determinant OR determinants OR association OR associations OR prognosis OR prognostic OR progression OR survival OR mortality)
```

Deduplication summary: 2,110 records identified; 431 duplicate records removed; 1,679 unique records entered screening.
